# Prophylactic Antioxidant Potential of Gallic Acid in Murine Model of Sepsis

**DOI:** 10.1155/2014/580320

**Published:** 2014-06-11

**Authors:** Harikesh Maurya, Vaishali Mangal, Sanjay Gandhi, Kathiresan Prabhu, Kathiresan Ponnudurai

**Affiliations:** ^1^Siddhartha Institute of Pharmacy, Near I.T. Park, Sahastradhara Road, Dehradun, India; ^2^City Heart Centre, 19 Cross Road, Dehradun 248001, India; ^3^Nova College Pharmacy, Vegavaram, Jangareddigudem, Andhra Pradesh 534447, India

## Abstract

Present study is to investigate the effect of Gallic acid pretreatment on survival of septic animals and oxidative stress in different organs like lungs, liver, kidney, spleen, and vascular dysfunction of mice. Sepsis was induced by cecal ligation and puncture (CLP) in healthy adult male albino mice (25–30 g) and was divided into 3 groups each consisting of 6 animals, that is, sham-operated (SO group (Group I), SO + sepsis (Group II), and Gallic acid + sepsis (Group III)). Group III animals were pretreated with Gallic acid at the dose rate of 20 mg/kg body weight for 2 days before induction of sepsis. Animals were sacrificed on 8th day and the tissue samples were obtained for further investigation on lipid peroxidation (LPO), malondialdehyde (MDA), superoxide dismutase (SOD), catalase (CAT), and glutathione reductase (GSH). Gallic acid pretreatment significant (*P* < 0.05) reduces kidney, spleen, liver, and lungs' malondialdehyde level in septic mice. However, it fails to improve reduced glutathione level in all given organs, while, Gallic acid pretreated mice showed significant improvement in SOD activity of kidney and spleen when compared to septic mice. Finally, the beneficial effects of Gallic acid pretreatment in sepsis are evident from the observations that Gallic acid partially restored SOD and catalase activity and completely reversed lipid peroxidation. Further studies are required to find out the possible mechanisms underlying the beneficial effects of Gallic acid on large population.

## 1. Introduction

Sepsis is multiple organ dysfunction syndromes, a leading cause for morbidity and mortality worldwide [1]. Its basic pathogenesis involves free radicals intervention; these radicals can react with cell component, leading to lipoperoxidation with loss of membrane fluidity creating a state of multiple organ failure [2–5]. Immunosuppressive and antimicrobial resistance patients are more prone to develop the sepsis [6]. Uncontrolled hyperinflammatory response and inappropriate cytokine response lead to early stages of sepsis. Hence, it is necessary to control inflammation during early stages of sepsis and to avoid the incidences of organ injury which may lead to organ failure and death. Further, it is reported that the activated leukocytes can produce reactive oxygen species (ROS) [7, 8] and disproportionate activation of respiratory burst involved in many diseases like asthma, diabetes, Parkinson's, and Alzheimer's disease [9–11] may be responsible for sepsis.

Both gram negative (*Pseudomonas aeruginosa*,* Klebsiella pneumoniae*, etc.) and gram positive (*Escherichia coli, Staphylococcus aureus,* etc.) organism can cause sepsis [12, 13]. Further, neuroimmunopathogenesis of sepsis involves peripheral release of proinflammatory cytokines (TNF-*α*, IL-1*α*, IL-1*β*, and IL-6) increasing blood brain barrier (BBB) permeability, generating neuroinflammation and sepsis associated sickness behaviour [14–16].

In recent decades the reported incidence of sepsis has increased considerably; due to the advancing age of the population, an increased number of invasive procedures performed along with immunosuppressive therapy [17]. Approximately 750,000 cases of sepsis occur each year, out of which almost 225,000 cases are fatal in United States alone [18, 19]. Gallic acid is related to phytochemical compounds found widely distributed in many plant tissues, both as a free compound and as a component of the plant polymer tannin. Gallic acid and its Catechin derived from its polymer tannin are present as main phenolic compounds of both black and green tea. This phenolic compound is possessing antioxidant property and appears to have antimicrobial and anticancer activities [20].

The present study was designed to decipher the role of Gallic acid pretreatment on survival of septic animals which may reduce inflammatory processes, organ injury, and death during early sepsis induced by cecal ligation and puncture (CLP). CLP shows progressive systemic inflammatory syndrome followed by septic shock and multiorgan injury in mice [21]. CLP model shows a cytokine profile similar to that in human sepsis and development of fulminant multiple organ failure [22].

## 2. Materials and Methods

### 2.1. Procurement of Gallic Acid and Reagents

Gallic acid was obtained as a gift sample from Natural Remedies Pvt. Ltd., Bangalore, India. All other chemicals and biochemical reagents were of L.R. and A.R. grade, respectively.

### 2.2. Experimental Animals

Healthy adult male albino mice (25–30 g) were obtained from Siddhartha Institute of Pharmacy, Dehradun, India. They were housed in groups of six under standard laboratory conditions of temperature (25 ± 2°C) and 12/12 h light/dark cycle. Animals had free access to standard pellet diet and water ad libitum. The distribution of animals in the groups, the sequence of trials, and the treatment allotted to each group were randomized throughout the experiment. Laboratory animal handling and experimental procedures were performed in accordance with the guidelines of Committee for the Purpose of Control and Supervision of Experiments on Animals (CPCSEA) and experimental protocol was approved by Institutional Animal Ethical Committee.

### 2.3. Surgical Details

Experimental mice were fasted overnight before the induction of sepsis. They were anesthetized by an intraperitoneal injection of xylazine 10 *μ*g/g and ketamine 80 *μ*g/g body weights. Animals were allowed to breath spontaneously during the surgery. A heating lamp was used to preserve the body temperature at approximately 37°C. Peritonitis was induced by CLP as described previously by Wichterman et al. (1980) [23, 24].

A 2 cm ventral midline incision was performed on the animals under anaesthesia and the cecum was exposed. A distended portion of the cecum just distal to the ileocecal valve was isolated, filled with fecal content and tied with a 3-0 silk suture in a manner that would not disrupt bowel continuity. The ligated portion of the cecum was punctured twice with a 21-gauge needle to avoid intestinal obstruction and a small amount of stool was expelled from the punctures to ensure leakage of the intestinal content. The cecum was replaced in its original position within the abdomen, the abdomen was closed with a 3-0 suture in 2 layers, and the animals were allowed to recover and normal saline (1 mL/mouse) was given subcutaneously to all mice to prevent dehydration. The SO group mice had undergone the same surgical procedure except cecal ligation and puncture [25–27].

After 24 hours of CLP, the abdomen was reopened and the ligated cecum was excised. Samples were taken by swabs for verification of the induced peritonitis. Peritoneal lavage was further continued with 40 mL of warm sterile saline. The left colon was transected from 3 to 4 cm above the peritoneal reflection, an end-to-end anastomosis was performed by using 1 layer of interrupted 7-0 sutures, and then the abdomen was closed primarily in all animals [28].

### 2.4. Study Groups

Animals were divided into 3 groups, with six animals in each group. In Group I (SO group) ceacum was ligated without puncture. In Group II (SO + sepsis), ceacum was ligated with puncture and then sutured. Surgery was performed by CLP. After the CLP surgery of group first and group second animals, they were kept for observation. Group III (Gallic acid + sepsis) animals were pretreated with Gallic acid at the dose rate of 20 mg/kg body weight for 2 days before induction of sepsis. Animals were fed with standard mice chow and water postoperatively [29].

### 2.5. Survival Study

Sepsis was induced through CLP, with SO group animals serving as controls. In preliminary experiments, we observed a hyperdynamic physiological state followed by a hypodynamic physiological state, similar to hemodynamic alterations in septic patients. It was thus decided to initiate Gallic acid therapy 2 days before sepsis induction by CLP. Mice were treated with Gallic acid or placebo. The degree of sepsis induced by CLP in mice was assessed by the presence of conjunctivitis, absence of grooming activities with resulting ruffled fur, no oral uptake of food or water, and lethargy. Survival curve and mean survival time for all the groups were performed by Kaplan-Meier survival curve and analyzed using log rank test [24].

### 2.6. Tissue Preparation

#### 2.6.1. Preparation of Liver Homogenate

A 200 mg of liver tissue was weighed and taken in 2 mL of ice-cold PBS (pH 7.4). Another 200 mg of sample was weighed and taken in 2 mL of 0.02 M ethylene diamine tetra acetic acid (EDTA) solution for reduced glutathione (GSH) estimation. The homogenate (10%) prepared with IKA homogenizer (Germany) under ice-cold condition was centrifuged for 10 min at 3000 rpm. The supernatant was stored at 20°C until assay of different oxidative stress-related biochemical parameters. A double beam UV-VIS Spectrophotometer (UV 5704SS, ECIL, India) was used for recording the absorbance of test samples [30].

#### 2.6.2. Preparation of Kidney Homogenate

A small portion of kidney tissue were immediately removed, weighed, minced, and homogenized (10%, w/v) separately in ice-cold 1.15% KCl-0.01 M sodium, potassium phosphate buffer (pH 7.4) in a Potter-Elvehjem type homogenizer. The homogenate was centrifuged at 18,000 ×g for 20 min at 4°C, and the resultant supernatant was used for subsequent biochemical analyses [31].

#### 2.6.3. Preparation of Lungs and Spleen Homogenate

Lungs and spleen were quickly removed and washed in ice-cold phosphate buffer saline (PBS). Washed tissues were immediately immersed in liquid nitrogen and stored at −70°C until biochemical analysis. On the day of use, frozen tissue samples were quickly weighed and homogenized 1 : 10 in ice-cold PBS. The homogenates were then centrifuged at 14,000 ×g for 15 min at 4°C. The supernatants were separated and used for enzyme activities [32].

### 2.7. Biochemical Estimation

#### 2.7.1. Lipid Peroxidation (LPO) in Different Tissues

The extent of lipid peroxidation was evaluated in terms of MDA (malondialdehyde) production determined by the thiobarbituric acid (TBA) method [33].

#### 2.7.2. Assessment of Antioxidant Eminence

*(a) Superoxide Dismutase*. Superoxide dismutase (SOD) was estimated as per the method described by Madesh and Balasubramanian [34]. It involves the generation of superoxide by pyrogallol autooxidation and inhibition of superoxide dependent reduction of the tetrazolium dye MTT [3-(4-5 dimethylthiazol 2-yl) 2,5-diphenyltetrazolium bromide] to its formazan, which is measured at 570 nm. The reaction was ended by the addition of dimethyl sulfoxide (DMSO), which helps to solubilize the formazan formed. The colour evolved was stable for many hrs and was expressed as SOD units (one unit of SOD is the amount of (*μ*g) protein required to inhibit the MTT reduction by 50%) [35, 36].

*(b) Catalase*. Catalase was assayed by spectrophotometric method as described by Aebi [37]. 10% tissue homogenate was used for estimation of catalase activity.

*(c) Reduced Glutathione (GSH)*. GSH was determined by estimating free-SH groups, using 5-5′ dithiobis 2-nitrobenzoic acid (DTNB) method of Sedlak and Lindsay [38]. 10% tissue homogenate was made in 0.02 M EDTA for estimation of GSH level.

#### 2.7.3. Estimation of Gallic Acid Pretreatment in Ach Induced Relaxations in Endothelium Intact Mouse Aorta

Thoracic aorta was cut into rings of 3-4 mm length. These aortic rings were mounted between two hooks made from 37 gauge stainless steel wire and were kept under a resting tension of 1.0 g in a thermostatically controlled (37.0 ± 0.1°C) organ bath (UGO Basile, Italy) of 10 mL capacity containing Modified Krebs-Henseleit solution (MKHS) and were continuously bubbled with medical gas (74% N_2_ + 21% O_2_ + 5% CO_2_). The aortic rings were equilibrated for 60–80 min in organ bath filled with MKHS before recording tension. During equilibration period, the bath fluid was repeatedly changed once in every 15 min. The change in tension was measured by a high-sensitivity isometric force transducer and recorded in a PC using Lab Chart V6.1.3 Pro software programme (Powerlab, AD Instruments, Australia).

After equilibration, aortic rings were contracted with high K^+^ (80 mM) depolarizing solution. On attaining contraction-plateau, high potassium solution was replaced by normal MKHS. The preparations were washed with normal MKHS to restore baseline resting tension [39]. Following a lapse of 30 min and 2-3 washes with normal MKHS, aortic rings were again contracted with phenylephrine and concentration-response curves to Ach were elicited by its cumulative addition to the bath solution at an increment of 0.5 log unit at plateau phase of agonist-induced contraction.

### 2.8. Statistical Analysis

Relaxation responses were expressed as the percentage reversal of the phenylephrine contraction. Both *E*_max⁡_ (the maximal response) and EC_50_ (the concentration producing 50% of the maximal response) were determined by nonlinear regression analysis (sigmoidal dose-response with variable slope) using Graph Pad Prism version 5.00 (San Diego, California, USA). Sensitivity/potency was expressed as pD_2_ = − log⁡⁡EC_50_. Results were expressed as mean ± S.E. with *n* equal to number of animals. Data were analyzed by Student-Newman-Keuls method for multiple group analysis. Survival percent was estimated by Kaplan-Meier method and compared by log rank test. Concentration-dependent agonist response data were analyzed by two-way ANOVA followed by Bonferroni post hoc test. Differences in values were considered statistically significant at *P* < 0.05 (Snedecor and Cochran).

## 3. Results

### 3.1. Assessment of Gallic Acid Pretreatment in Sepsis on Survival Time in Mice

**Figure 1:**
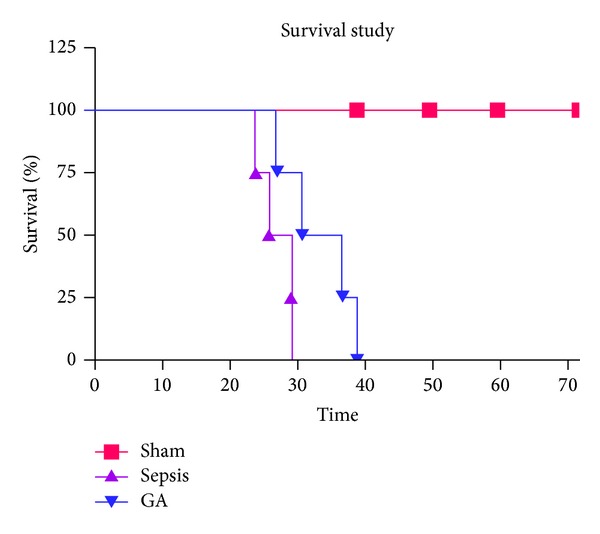
Effect of Gallic acid pretreatment in sepsis on survival time in mice.

The results denote mean survival time for sepsis (treated with vehicle) and pretreatment with Gallic acid (20 mg/kg B.W. orally) was 27 : 10 ± 1 : 26 h and 38 : 08 ± 1 : 80 h, respectively (Figure 1). Thus, Gallic acid shows significant (*P* < 0.05) effect on the survival time in comparison to the septic mice (group II). All the sham-operated mice (group I) survived during 72 : 00 h observation period.

### 3.2. Effect of Gallic Acid Pretreatment in Sepsis on Lipid Peroxidation Level (nmol/mg) in Different Organs of Mice

The extent of lipid peroxidation was evaluated in terms of MDA (malondialdehyde) production determined by the thiobarbituric acid (TBA) method (Table 1).

*Kidney Tissue*. The effect of Gallic acid pretreatment in sepsis on kidney MDA level was found to be 5.87 ± 0.82 nmol/mg in sham-operated (SO group) treatment group. In comparison to this, the sepsis treatment group shows the significant (*P* < 0.05) increased level of MDA to 8.98 ± 0.79 nmol/mg. However, Gallic acid pretreatment significantly (*P* < 0.05) reduced kidney MDA level to 7.06 ± 0.78 nmol/mg in septic mice.

*Spleen Tissue*. The effect of Gallic acid pretreatment in sepsis on spleen MDA level was found to be 7.50 ± 0.80 nmol/mg in SO treatment group. In comparison to this, the sepsis treatment group shows the significant (*P* < 0.05) increased level of MDA to 11.00 ± 0.48 nmol/mg. However, Gallic acid pretreatment significantly (*P* < 0.05) reduced spleen MDA level to 8.00 ± 0.85 nmol/mg in septic mice.

*Liver Tissue*. The effect of Gallic acid pretreatment in sepsis on liver MDA level was found to be 12.68 ± 0.92 nmol/mg in SO treatment group. In comparison to this, the sepsis treatment group shows the significant (*P* < 0.05) increased level of MDA to 36.02 ± 0.62 nmol/mg. However, Gallic acid pretreatment significantly (*P* < 0.05) reduced liver MDA level to 18.96 ± 0.88 nmol/mg in septic mice.

*Lungs Tissue*. The effect of Gallic acid pretreatment in sepsis on lungs MDA level was found to be 4.22 ± 0.75 nmol/mg in SO treatment group. In comparison to this, the sepsis treatment group shows the significant (*P* < 0.05) increased level of MDA to 9.06 ± 0.80 nmol/mg. However, Gallic acid pretreatment significantly (*P* < 0.05) reduced lungs MDA level to 6.21 ± 0.68 nmol/mg in septic mice.

### 3.3. Effect of Gallic Acid Pretreatment in Sepsis on Superoxide Dismutase (SOD Units/mg) Activity in Different Organs of Mice

The SOD level was evaluated in different tissues as mentioned in Table 2.

*Kidney Tissue*. The SOD activity in kidney tissue shows significant reduction in sepsis (Group II) which was 23.80 ± 2.06 units/mg as in comparison to SO treatment group, that is, 33.68 ± 1.08 units/mg. Gallic acid pretreated mice (group III) showed significant improvement in SOD activity, 57.46 ± 1.38 units/mg, as compared to septic mice.

*Spleen Tissue*. The level of SOD activity in septic mice (Group II) 10.22 ± 1.78 units/mg was significantly lower than SO treatment group, that is, 19 ± 1.6 units/mg. Gallic acid pretreated mice (Group III) did not show significant change in SOD activity (13.21 ± 1.55 units/mg) as compared to group II.

*Liver Tissue*. Significant reduction in SOD activity found in septic mice was 25.01 ± 1.62 units/mg as compared to SO treatment group, that is, 58.12 ± 2.00 units/mg. Gallic acid pretreatment was not able to reverse the SOD activity (25.01 ± 1.62 units/mg).

*Lungs Tissue*. There was significant decrease in SOD activity (20.48 ± 1.70 units/mg) in group II as compared to SOD activity (42.63 ± 1.66 units/mg) with group I. Gallic acid pretreatment reversed the SOD activity to 51.33 ± 1.54 units/mg.

### 3.4. Effect of Gallic Acid Pretreatment in Sepsis on Catalase (CAT) Activity (mmol H_2_O_2_/min/mg) in Different Organs of Mice

The catalase activity was performed on different organs after Gallic acid pretreatment. The results are mentioned in Table 3.

*Kidney Tissue*. The change of catalase activity in sepsis treatment group was 0.33 ± 0.03 mM H_2_O_2_/min/mg as compared to SO treatment group, that is, 0.72 ± 0.04 mM H_2_O_2_/min/mg. Gallic acid pretreatment did not show any significant change in catalase activity 0.54 ± 0.03 mM H_2_O_2_/min/mg as compared to septic mice.

*Spleen Tissue*. Group II animals did not show significant change of catalase activity 0.27 ± 0.01 mM H_2_O_2_/min/mg in comparison to group I treatment animals 0.32 ± 0.02 mM H_2_O_2_/min/mg, while Gallic acid pretreatment shows significant increase in catalase activity 0.42 ± 0.04 mM H_2_O_2_/min/mg in comparison to group I animals.

*Liver Tissue*. In this, there was no significant difference observed in catalase activity of group II animals 0.46 ± 0.05 mM H_2_O_2_/min/mg as compared to group I animals 0.66 ± 0.04 mM H_2_O_2_/min/mg. Gallic acid pretreated mice also did not show any significant change in catalase activity 0.35 ± 0.03 mM H_2_O_2_/min/mg as compared to septic mice.

*Lungs Tissue*. Septic mice did not show significant decrease in catalase activity 0.38 ± 0.02 mM H_2_O_2_/min/mg as compared to SO mice 0.52 ± 0.05 mM H_2_O_2_/min/mg. Gallic acid pretreatment did not reverse significant change in catalase activity 0.55 ± 0.02 mM H_2_O_2_/min/mg as compared to septic mice.

### 3.5. Effect of Gallic Acid Pretreatment in Sepsis on Reduced Glutathione Level (mM GSH/g Wet Tissue) in Liver of Mice

The effect of Gallic acid pretreatment in sepsis on reduced GSH level in liver tissue was determined in Table 4. Sepsis significantly decreased the reduced GSH level (0.46 ± 0.04 mM GSH/g) of wet tissue as compared to SO mice 0.85 ± 0.06 mM GSH/g wet tissue. However, Gallic acid pretreatment in sepsis failed to improve reduced glutathione level (0.65 ± 0.05 mM GSH/g wet tissue).

### 3.6. Effect of Gallic Acid Pretreatment in Sepsis on Ach Induced Relaxations in Endothelium Intact Mouse Aorta

PE (1 *μ*M) precontracted endothelium intact rings from aorta of SO mice exhibited concentration-dependent relaxations to Ach (0.1 Nm–10 *μ*M) with pD_2_ and *E*_max⁡_ of 06.96 ± 0.07 and 93.55 ± 2.84%, respectively, as given in Table 5. Sepsis significantly (*P* < 0.05) reduced the concentration dependent relaxation to Ach with pD_2_ and *E*_max⁡_ of 06.09 ± 0.19 and 72.72 ± 5.67%, respectively, and also did not uphold the relaxation to Ach (*E*_max⁡_ and pD_2_) in sepsis.

## 4. Discussion and Conclusion

Sepsis, inflammation, and particularly septic shock are associated with global and local hypoperfusion, ischemia-reperfusion events, endothelial injury with an associated procoagulant state, and monocyte-macrophage system activation. They also induce the production of large amounts of free radicals in a nonregulated fashion associated with high-oxidative potential damage. In fact, several sources of reactive oxygen species (ROS) have been detected in sepsis and septic shock, including the mitochondrial respiratory electron transport chain, immune cell, and xanthine oxidase activation as a result of ischemia and reperfusion and the respiratory burst associated with NADPH oxidase. Several studies have shown the presence of oxidative stress in sepsis. Early production of reactive oxygen species (ROS) has been demonstrated in experimental studies in sepsis and excessive release of superoxide anion has been shown to contribute to postreperfusion oxidative damage in several ischemic organs. During sepsis, activation of proinflammatory pathways leads to dysfunction of mitochondria and cells, which contributes to multiorgan failure and poor outcomes. Proinflammatory cytokines like TNF-*α* increase cytosolic levels of Ca^2+^ via inositol-1,4,5-triphosphate-mediated (IP_3_-mediated) pathway from the ER. The increase in cytosolic Ca^2+^ is followed by a rapid increase in mitochondrial Ca^2+^, leading to a rise in mitochondrial ROS generation from complex III of the electron transport chain. Higher level of ROS results in the release of cytochrome* c* and cell death.

Gallic acid and its structurally related compounds are found widely distributed in fruits and plants. Studies utilising these compounds have found them to possess many potential therapeutic properties including anti-cancer and antimicrobial properties. In addition to direct antioxidant activity, Gallic acid and its derivatives may function indirectly as an antioxidant by enhancing antioxidant enzymes, such as heme oxygenase, glutathione peroxidase, glutathione reductase, and catalase, and phase-2 enzymes such as glutathione S transferase and quinone reductase. In Gallic acid pretreatment group, we studied the role of Gallic acid against sepsis induced oxidative stress in different organs of albino mice. Gallic acid showed protective role by decreasing the LPO level in animals exposed to CLP. It worked as an antioxidant and increased the enzymatic antioxidants CAT and SOD in animals exposed to CLP. The organ specific effect may be further studied to reach at any conclusion. The reduction of oxidative stress in the test animals may be by scavenging ROS, protecting the antioxidant enzymes from being denatured, and reducing the oxidative stress marker LPO. A study has reported the improvements of haemodynamics and vascular response by Gallic acid pre- and posttreatment during endotoxemia (LPS model) in which they have attributed to alleviation of oxidative stress by reducing aortic-derived superoxide production, suppression of lipid peroxidation and protein oxidation, and decrease in urinary nitric oxide metabolites with preservation of the ratio of glutathione/glutathione disulfide. Similar to their observation, current study showed suppression of lipid peroxidation in all the tissues under study in CLP model of polymicrobial sepsis. In contrast to their study, we did not find significant improvement in reduced glutathione content in liver and SOD activity in spleen and liver [40].

In the present study, the elevated level of LPO in CLP model of mice may be due to oxidative stress and enhanced reactive oxygen species (ROS) production by the inflammatory process. In accordance with the results of previous study, we have found increased MDA production in all the tissues after 18 hrs of sepsis, which is also supportive of the clinical study [41, 42]. In an older study, the authors have reported the increased thiobarbituric acid reactive substances (TBARSs), which are a marker of lipoperoxidation, in critically ill patients in association with multiorgan failure (MOF) development [43].

In conclusion, the beneficial effects of Gallic acid pretreatment in sepsis are evident from the observations that Gallic acid partially restored SOD and catalase activity and completely reversed lipid peroxidation. Further studies are required to find out the possible mechanisms underlying the beneficial effects of Gallic acid. It is integrated that the Gallic acid can be exploited in the treatment of inflammatory disease (sepsis), on the basis of results and outcomes of present research work.

## Figures and Tables

**Table 1:** Effect of Gallic acid pretreatment in sepsis on lipid peroxidation level (nmol/mg) in different organs of mice.

Sr. number	Number of groups	Kidney tissue	Spleen tissue	Liver tissue	Lungs tissue
1	Group I	05.87 ± 0.82	07.50 ± 0.80	12.68 ± 0.92	04.22 ± 0.75
2	Group II	08.98 ± 0.79*	11.00 ± 0.48*	36.02 ± 0.62*	09.06 ± 0.80*
3	Group III	07.06 ± 0.78^#^	08.00 ± 0.85^#^	18.96 ± 0.88^#^	06.21 ± 0.68^#^

*P < 0.05 shows statistically significant difference in comparison to first group.

^#^ *P* < 0.05 shows statistically significant difference in comparison to second group.

**Table 2:** Effect of Gallic acid pre-treatment in sepsis on superoxide dismutase (SOD units/mg) activity in different organs of mice.

Sr. number	Number of groups	Kidney tissue	Spleen tissue	Liver tissue	Lungs tissue
1	Group I	33.68 ± 1.08	21.04 ± 1.60	58.12 ± 2.00	42.63 ± 1.66
2	Group II	23.80 ± 2.06*	10.22 ± 1.78*	25.01 ± 1.62*	20.48 ± 1.70*
3	Group III	57.46 ± 1.38^#^	13.21 ± 1.55	30.86 ± 1.80	51.33 ± 1.54^#^

*P < 0.05 shows statistically significant difference in comparison to first group.

^#^ *P* < 0.05 shows statistically significant difference in comparison to second group.

**Table 3:** Effect of Gallic acid pretreatment in sepsis on catalase (CAT) activity (mmol H_2_O_2_/min/mg) in different organs of mice.

Sr. number	Number of groups	Kidney tissue	Spleen tissue	Liver tissue	Lungs tissue
1	Group I	0.72 ± 0.04	0.32 ± 0.02	0.66 ± 0.04	0.52 ± 0.02
2	Group II	0.33 ± 0.03*	0.27 ± 0.01	0.46 ± 0.05*	0.38 ± 0.02
3	Group III	0.54 ± 0.03^#^	0.42 ± 0.04^#^	0.35 ± 0.03	0.55 ± 0.02^#^

*P < 0.05 shows statistically significant difference in comparison to first group.

^#^ *P* < 0.05 shows statistically significant difference in comparison to second group.

**Table 4:** Effect of Gallic acid pretreatment in sepsis on reduced glutathione level (mM GSH/g wet tissue) in liver of mice.

Sr. number	Number of groups	Liver tissue
1	Group I	0.85 ± 0.06
2	Group II	0.46 ± 0.04*
3	Group III	0.65 ± 0.05^#^

*P < 0.05 shows statistically significant difference in comparison to SO group.

^#^ *P* < 0.05 shows statistically significant difference in comparison to sepsis.

**Table 5:** Effect of Gallic acid pretreatment in sepsis on Ach induced relaxations in endothelium intact mouse aorta.

Sr. number	Number of groups	pD_2_	*E*_max⁡_ (%)
1	Group I	06.96 ± 0.07	93.55 ± 2.84
2	Group II	06.09 ± 0.19	72.72 ± 5.67*
3	Group III	07.39 ± 0.43	72.97 ± 8.59*

*P < 0.01 shows statistically significant difference as comparison to SO group.
